# Complete fourier direct magnetic resonance imaging (CFD-MRI) for diffusion MRI

**DOI:** 10.3389/fnint.2013.00018

**Published:** 2013-04-02

**Authors:** Alpay Özcan

**Affiliations:** Health Research, Arlington Innovation Center, Virginia Polytechnic Institute and State UniversityArlington, VA, USA

**Keywords:** magnetic resonance imaging, diffusion weighted imaging, fourier transform

## Abstract

The foundation for an accurate and unifying Fourier-based theory of diffusion weighted magnetic resonance imaging (DW–MRI) is constructed by carefully re-examining the first principles of DW–MRI signal formation and deriving its mathematical model from scratch. The derivations are specifically obtained for DW–MRI signal by including all of its elements (e.g., imaging gradients) using complex values. Particle methods are utilized in contrast to conventional partial differential equations approach. The signal is shown to be the Fourier transform of the joint distribution of number of the magnetic moments (at a given location at the initial time) and magnetic moment displacement integrals. In effect, the *k*-space is augmented by three more dimensions, corresponding to the frequency variables dual to displacement integral vectors. The joint distribution function is recovered by applying the Fourier transform to the complete high-dimensional data set. In the process, to obtain a physically meaningful real valued distribution function, phase corrections are applied for the re-establishment of Hermitian symmetry in the signal. Consequently, the method is fully unconstrained and directly presents the distribution of displacement integrals without any assumptions such as symmetry or Markovian property. The joint distribution function is visualized with isosurfaces, which describe the displacement integrals, overlaid on the distribution map of the number of magnetic moments with low mobility. The model provides an accurate description of the molecular motion measurements via DW–MRI. The improvement of the characterization of tissue microstructure leads to a better localization, detection and assessment of biological properties such as white matter integrity. The results are demonstrated on the experimental data obtained from an *ex vivo* baboon brain.

## 1. Introduction

Since the conception of mathematical models for the effect of the magnetic moment diffusion in nuclear magnetic resonance (NMR) experiments by Hahn (1950), Carrand Purcell (1954), and Torrey (1956), several methods have been proposed for analysis of diffusion-weighted (DW) magnetic resonance imaging (MRI) signal. These advancements endowed with the non-invasive, *in vivo* nature of the technique, have propelled the initial utilization of DW imaging measures, e.g., apparent diffusion coefficient in early detection of ischemia (Moseley et al., 1990; Baird and Warach, 1998), to many highly crucial areas in research and clinical imaging: for example in cancer diagnosis (Song et al., 2002; Turkbey et al., 2009; Xu et al., 2009), follow-up on treatment, pre- and post-operative assessment for different organs [e.g., fiber tracking (Conturo et al., 1999; Mori and van Zijl, 2002)] white matter integrity assessment (Budde et al., 2007; Correia et al., 2008) as in monitoring of neurological diseases such as multiple sclerosis (Song et al., 2002) and disorders (Ciccarelli et al., 2008) like schizophrenia (Seal et al., 2008; Voineskos et al., 2010) and Alzheimer's disease (Mielke et al., 2009), as well as neonatal development (McKinstry et al., 2002) and traumatic brain injury (Mac Donald et al., 2011).

In brief, diffusion weighted magnetic resonance imaging (DW–MRI) has become an indispensable and versatile technique playing an important role in several applications by its ability to estimate diffusion. The abundance of DW–MRI models is an indicator of room for improvement as well as the necessity for unification [see Özcan et al. (2012) for a detailed account of the partial differential equation (PDE) based adaptation's implications as well as a thorough mathematical analysis and a description of the background of existing methods].

DW–MRI's aim is to obtain measures and characterization of microstructure by investigating the diffusion process. Several methods and models have been proposed, all originating from the seminal work of Stejskal and Tanner (1965). Therein, under the influence of the additional motion sensitizing magnetic field gradients, the self-diffusion PDE of the magnetic moments is included in the Bloch PDE to model the attenuation in the DW–NMR spectroscopy signal. The result is the estimation of the *scalar* diffusion coefficient of the entire sample. In a sense, DW–NMR added another dimension, i.e., the *magnetic moment motion*, to the spectroscopic information even before the introduction of *magnetic moment position* later by the invention of MR imaging.

Accordingly, herein, DW–MRI model is naturally progressed to a higher dimensional construct that jointly presents magnetic moment *position* and *motion*. This is achieved by carefully re-examining the first principles of DW–MRI signal formation using particle methods in the spirit of the work of McCall et al. (1963). The mathematical model constructed in section 2.1 is specifically obtained for DW–MRI signal (rather than DW–NMR) by including all of its elements (e.g., imaging gradients) using complex values without taking the signal's magnitude.

The approach reveals that for an *l*_mr_-dimensional MRI slice, the DW–MRI complex valued signal that comes out of the scanner is the (*l*_mr_ + 3)-dimensional Fourier transform of the *joint distribution function* of the number of magnetic moments (that are at a given position at the initial time) and their displacement integrals. In other words, the first *l*_mr_ dimensions correspond to the usual MRI *k*-space with position information and the remaining three dimensions constitute the frequency space of displacement integrals. The values of imaging and motion sensitizing magnetic field gradient vectors together define in the (*l*_mr_ + 3)-dimensional Fourier space the sampling points of the joint distribution function's Fourier transform. The distribution function is recovered by taking the Fourier transform of the *complete* data *directly* (i.e., without any scaling or use of magnitudes), giving the method its name: Complete Fourier Direct (CFD) MRI (Özcan 2010b).

## 2. Materials and methods

### 2.1. Complete fourier direct MRI signal formation

The MR signal is generated by the vectorial sum of transverse magnetization of magnetic moments (Haacke et al., 1999):

(1) $$M(t)=\sum_{i}m_{i}(t).$$

By neglecting the effect of spin–spin relaxation, the evolution of the transverse magnetization of the *i*th magnetic moment is described in a standard manner by a rotating magnetization vector according to Bloch equations (see Appendix B): 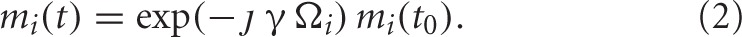


Here, γ is the gyromagnetic ratio, the transverse magnetization vector, *m*_*i*_, is written in complex number form with *m*_*i*_(*t*_0_) denoting the initial magnetization tipped to the transverse plane,

(3) $$\Omega_{i}=\int_{t_{0}}^{t}G(x_{i},\;\tau )\;\cdot \;x_{i}(\tau )d\tau$$

describes the phase (when multiplied by γ) as a function of the magnetic field gradients $G(x,t)\in {\mathbb{R}}^{3}$, and the position of the magnetic moment is $x_{i}\in {\mathbb{R}}^{3}$. The time-dependent position, *x*_*i*_, in Equation (3) affects the phase, Ω_*i*_, thereby also affecting the total signal in Equation (1).

Any kind of displacement (such as Brownian motion, molecular movement in biological tissue with different medium and obstacles, coherent motion or any combination thereof) is incorporated straight into the model, by modeling the position in a general and direct form herein without any stochastic assumptions [such as Markovian property used in Wedeen et al. (2005)] on the motion

(4) $$x_{i}(t)=x_{i}(t_{0})+w_{i}(t),$$

where $w_{i}(t)\in {\mathbb{R}}^{3}$ represents the displacement of the magnetic moment from its initial position, *x*_*i*_(*t*_0_), [i.e., *w*_*i*_(*t*_0_) = 0]. The only physical requirement is the continuity of *w*_*i*_(*t*) since a magnetic moment cannot disappear at a given point and reappear at another.

**Figure 1:**
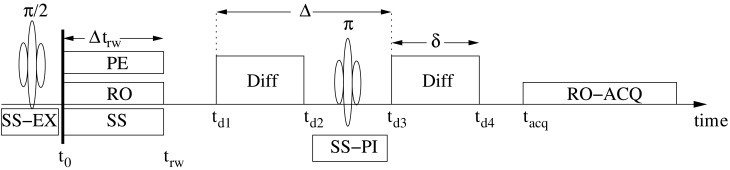
**The pulsed-gradient spin-echo (PGSE) pulse sequence and the definition of the variables used in the calculations.** SS–EX is for the slice select gradient during the excitation (π/2) pulse, RO for read out, PE for phase encode, SS for is the slice select gradient, Diff marks the motion sensitizing pulses, SS–PI is the slice select gradient during the π-pulse and ACQ stands for digital acquisition period. In practice, the MR pulse sequences implement the rewind (rw) gradients such that the amplifiers are turned on and off at the same times.

The signal is calculated using Equations (1– 4) in reverse order. Following the motion described by Equation (4), the phase of the *i*th magnetic moment in Equation (3) during the digital acquisition period of the two dimensional imaging (*l*_mr_ = 2) pulsed-gradient spin-echo (PGSE) sequence of Figure 1, is obtained after tedious but routine derivations [see Appendix B for a brief exposition of the derivations and Özcan (2012)] using the definitions of the variables in Figure 1 with $G_{*}\in {\mathbb{R}}^{3}$ denoting the magnetic field gradient vectors labeled as read-out, ro; phase encode, pe; slice select, ss; and diffusion, D. Omitting routine calculations for trapezoidal shapes for clarity, the derivation is carried out assuming ideal gradient amplifiers providing rectangular shaped gradient pulses. The initial time, *t*_0_, is the end time of π/2 radio frequency (RF) pulse when the magnetization is fully tipped to the transversal plane. The resulting phase of the transverse magnetization is a function of time, *t*, and, the imaging and diffusion gradients (see Appendix B):

(5) $$\Omega_{i}(t,\;G_{\text{pe}},\;G_{\text{ss}},\;G_{\text{D}})=\left((t-t_{\text{acq}}-\Delta t_{\text{rw}})\;G_{\text{ro}}-\Delta t_{\text{rw}}\;G_{\text{pe}}\right)\;\cdot \;x_{i}(t_{0})-\Delta t_{\text{rw}}\;G_{\text{ss}}\;\cdot \;x_{i}(t_{0})$$

(6) $$+G_{\text{D}}\;\cdot \;W^{\text{d}}+((t_{d4}-t_{d3})-(t_{d2}-t_{d1}))G_{\text{D}}\;\cdot \;x_{i}(t_{0})+\Phi_{\pi i}$$

(7) $$+G_{\text{ro}}\cdot \;W_{i}^{\text{acq}}(t)-(G_{\text{ro}}+G_{\text{pe}}+G_{\text{ss}})\;\cdot \;W_{i}^{\text{rw}}.$$

The second term in Equation (6) removes the injection of the initial position into the DW signal because of equal pulse duration times, δ = *t*_*d*4_ − *t*_*d*3_ = *t*_*d*2_ − *t*_*d*1_. The term Φ_π*i*_ describes the systematic phase (see Appendix B1) created by the π-pulse slice select gradient (SS–PI) and in this work it will be automatically taken out by the phase correction algorithm in section 2.2. Equations (6) and (7) incorporate three integrals of the displacement *w*_*i*_(*t*): (*W*^d^_*i*_, *W*^acq^_*i*_, *W*^rw^_*i*_) corresponding to the displacement integrals for diffusion (d), analog to digital conversion acquisition (acq), and initial rewind (rw) gradient time periods, respectively

(8) $$W_{i}^{\text{d}}=\int_{t_{d3}}^{t_{d4}}w_{i}(\tau )\;d\tau -\int_{t_{d1}}^{t_{d2}}w_{i}(\tau )\;d\tau ,$$

(9) $$W_{i}^{\text{acq}}(t)=\int_{t_{\text{acq}}}^{t}w_{i}(\tau )\;d\tau ,\;W_{i}^{\text{rw}}=\int_{t_{0}}^{t_{\text{rw}}}w_{i}(\tau )\;d\tau .$$

First term in Equation (5) is the definition of the *k*-space in regular MRI, $k_{\text{mr}}\in {\mathbb{R}}^{2}$:

(10) $$k_{\text{mr}}(t,\;G_{\text{pe}})=(t-t_{\text{acq}}-\Delta t_{\text{rw}})\;G_{\text{ro}}-\Delta t_{\text{rw}}\;G_{\text{pe}}$$

with the additional term,

(11) $$\varphi_{\text{slice}}=-\Delta t_{\text{rw}}\;G_{\text{ss}}\;\cdot \;x_{i}(t_{0}),$$

which is constant because the slice select axis component of *x*_*i*_(*t*_0_) is the slice position^1^.

Without loss of generality, by adopting the imaging coordinate frame defined by the directions of the read-out, phase encode and slice select gradients, *G*_ro_ = [*g*_ro1_, 0, 0], *G*_pe_ = [0, *g*_pe2_, 0], *G*_ss_ = [0, 0, *g*_ss3_], time and *g*_pe2_ become functions of *k*_mr_ = [*k*_mr1_, *k*_mr2_, *k*_mr3_] using Equation (10):

$$t=k_{\text{mr}1}/g_{\text{ro}1}+t_{\text{acq}}+\Delta t_{\text{rw}}\text{ and }g_{\text{pe2}}=-k_{\text{mr2}}/\Delta t_{\text{rw}}.$$

Accordingly, *W*^acq^_*i*_(*t*) becomes a function of *k*_mr1_ and the coefficients of *W*^rw^ in Equation (7) are written as a vector which is an *affine* function of *k*_mr_:

(12) $$k_{\text{rw}}=\left[-g_{\text{ro}1},\;k_{\text{mr}2}/\Delta t_{\text{rw}},\;-g_{\text{ss}3}\right].$$

Consequently, the phase, Ω_*i*_, of Equation (3) is expressed concisely by defining *k*_D_ = *G*_D_:

(13) $$\Omega_{i}=k_{\text{mr}}\;\cdot \;x_{i}(t_{0})+k_{\text{D}}\;\cdot \;W_{i}^{\text{d}}+k_{\text{rw}}\;\cdot \;W_{i}^{\text{rw}}+W_{i,\;1}^{\text{acq}}(k_{\text{mr}1})\;g_{\text{ro}1}+\varphi_{\text{slice}},$$

reflecting the effect of initial position and displacement integrals on the phase^2^ of each magnetic moment. Since ϕ_slice_ is constant for all *i*, it is taken out of Equation (13) with the appropriate rotation of the magnetization coordinate frame on a slice by slice basis.

Finally, assuming that all of the magnetic moments have the real valued initial magnetization *m*_*i*_(*t*_0_) = *m*_0_, Equation (1) can be re-written using Equation (13) to reveal a Fourier relationship, 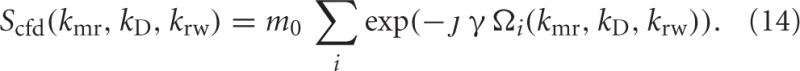


A more efficient way to evaluate the sum in Equation (14) is first to group the magnetic moments with the same position-displacement properties and then to count the numbers elements in the groups.

**Definition 1.** *The joint position-displacement integral distribution function, *P*^total^_cfd_(*x*, *W*), is defined as the number of magnetic moments with the initial position $x\in {\mathbb{R}}^{3}$ at time *t*_0_, possessing the displacement integral values of $W=(W^{\text{d}},W^{\text{rw}},W_{1}^{\text{acq}})\in {\mathbb{R}}^{7}$.*

The signal in Equation (14) is calculated by integrating over the whole position-displacement space (absorbing *m*_0_ into *P*^total^_cfd_ for ease of notation): 
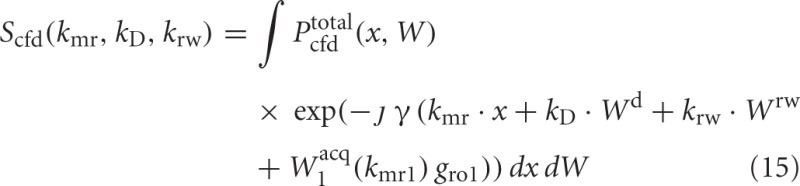

(16) $$=ℱ\{P_{\text{cfd}}^{\text{total}}\}(k_{\text{mr}},\;k_{\text{D}},\;k_{\text{rw}}).$$

Equation (15) is by definition the Fourier transform of *P*^total^_cfd_ with non-linearities added by *W*^acq^_1_.

Among the elements of *W*, the focus is on the most descriptive MRI observable, *W*^d^. Its distribution is obtained by marginalizing *W*^rw^ from Equation (15)

(17) $$\begin{array}{l} P_{\text{cfd}}(x,\;W^{\text{d}})=\int P_{\text{cfd}}^{\text{total}}(x,\;W^{\text{d}},\;W^{\text{rw}})\;dW^{\text{rw}}\\ \;={ℱ}_{\left(k_{\text{mr}},\;k_{\text{D}}\right)}^{-1}\{S_{\text{cfd}}(k_{\text{mr}},\;k_{\text{D}},\;0)\}. \end{array}$$

However, the affine dependence of *k*_rw_ in Equation (12) makes it impossible to fix *k*_rw_ = 0 and to sample in (*k*_mr_, *k*_D_, 0) subspace. The following example demonstrates how the affine dependence affects the measurements by using a two dimensional Gaussian function, exp(−(*k*^2^_1_ + *k*^2^_2_)), with the “undesirable” variable *k*_2_ sampled on a line *k*_2_ = *a k*_1_ + *b*: 
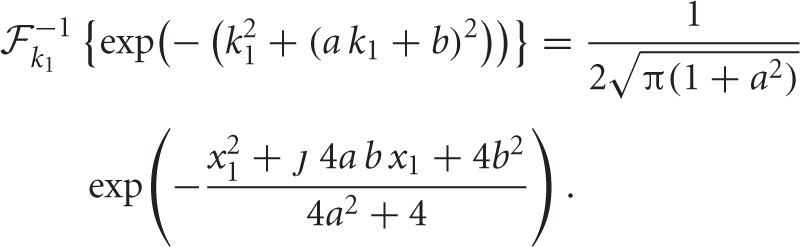


The result is complex valued in comparison to the real valued Fourier transform of exp(−*k*^2^_1_) which can be obtained by setting *a* = *b* = 0.

### 2.2. Phase corrections for the estimation of *P*_cfd_

In addition to inherent affine dependence and non-linearities, different experimental factors, noise, hardware imperfections etc., affect the DW–MR signal adversely. CFD–MRI addresses these issues by adopting a pivotal, physically meaningful standpoint originating from the following Fourier transform property (Bracewell, 2000):

(real valued function) ⇆ ℱ ⇆ (Hermitian symmetric function).

Accordingly CFD–MRI reconstruction is based on the following:

Since by definition *P*^total^_cfd_ is real valued, *S*_cfd_ is Hermitian symmetric.

Furthermore, an immediate implication of the transform property and the Hermitian symmetry of *S*_cfd_ is that theoretically, taking its magnitude before Fourier transforming will result in a symmetric real valued distribution under ideal conditions. As noted above, in practice the experimental *S*_cfd_ is never Hermitian symmetric resulting in an asymmetric magnitude. Consequently, the magnitude's Fourier transform used in existing methods (Callaghan, 1991; Wedeen et al., 2005), results in a complex valued (Hermitian symmetric) distribution function. The difficulty of a physical interpretation forced those methods to take the magnitude of the transform as well to obtain a real valued function.

Herein, in order to obtain a real valued *P*_cfd_, the re-establishment of Hermitian symmetry in the signal during the computation of the inverse transform for Equation (17), is realized by phase corrections. The strategy is similar in principle to the correction of the *k*_mr_-space center's (echo time) shift during the read-out period of acquisition in MR imaging. The resulting linear phase shift in the physical read-out axis uniformly and systematically appears in all of the data. The shifts in both phase-encode (e.g., due to sample shaking) and read-out directions are corrected by first determining from the Fourier transform in *k*_mr_,

(18) $$I_{k_{\text{mr}}}^{\text{complex}}((x_{\text{ro}},\;x_{\text{pe}}),\;k_{\text{D}})={ℱ}_{k_{\text{mr}}}^{-1}\{S_{\text{cfd}}(k_{\text{mr}},\;k_{\text{D}},\;k_{\text{rw}})\},$$

the angle (∠ *I*_*k*^complex^_mr__) from the signal regions along the center lines of each physical direction at *k*_D_ = 0,

(19) $$r_{\text{ro}}(x_{\text{ro}})\approx ∠I_{k_{\text{mr}}}^{\text{complex}}((x_{\text{ro}},\;0),\;0),\;r_{\text{pe}}(x_{\text{pe}})\approx ∠I_{k_{\text{mr}}}^{\text{complex}}((0,\;x_{\text{pe}}),\;0).$$

The phase corrections are then applied systematically at each value of *k*_D_ (see Figure 3): 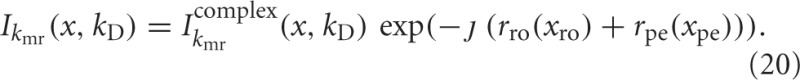


The Fourier transform in the remaining variables,

(21) $$P_{\text{cfd}}(x,\;W^{\text{d}})={ℱ}_{k_{\text{D}}}^{-1}\{I_{k_{\text{mr}}}(x,\;k_{\text{D}})\},$$

is evaluated sequentially in each *k*_D_-dimension with the aim of re-establishing the Hermitian property, *I*_*k*_mr__(*x*, −*k*_D_) = *I*_k^*^_mr__(*x*, *k*_D_), using the following steps.

**CFD Phase correction algorithm:**

**Figure 2:**
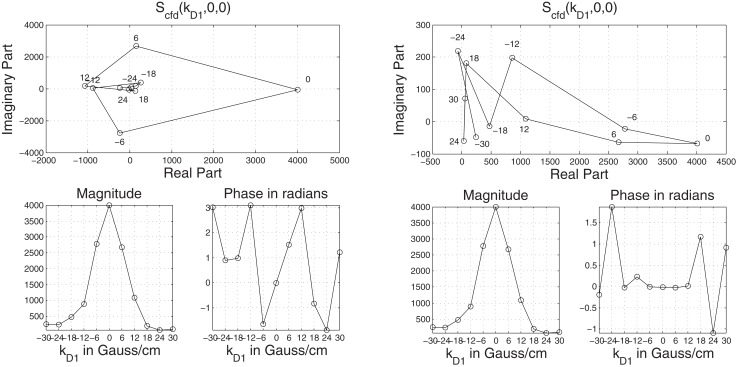
**Özcan (2012): **Top row**: The Nyquist plots of uncorrected (left) and corrected (right) *S*_cfd_ obtained from the experimental data described in section 2.4.** The plots show data acquired at each diffusion gradient value *k*_D1_ on the complex plane. **Bottom row**: The magnitude and phase plots of the data. Uncorrected data (bottom row left, second column) exhibit a linear phase shift around 0 frequency, indicative of coherent motion. After the phase corrections obtained using the polynomial 0.266*k*_D1_ estimated from the points *k*_D1_ = ‒6, 0, 6, 12 Gauss/cm, the magnitude is unchanged but the signal's imaginary part is smaller for the corrected values visible by the difference between the vertical axis spans of Nyquist plots and the phase plots.

1. Pick a pixel at location *x*_*c*_, preferably near the center of the image where tissue or a good signal area is located.
2. Starting from the first direction, *l* = 1 of the *k*_D_ space calculate the phase on the line passing through the origin (i.e., [*k*_D1_, 0, 0], [0, *k*_D2_, 0], [0, 0, *k*_D3_], respectively for *l* = 1, 2, 3), e.g., ∠*I*_*k*_mr__(*x*_*c*_,(*k*_D1_, 0, 0)).
3. Investigate the plot of the phase versus *k*_D*l*_. Pick as many as possible consecutive values of *k*_D*l*_ near 0 without sudden changes to assure high signal to noise value.
4. Construct a polynomial of degree *m* (with *m* less than the number of points) that approximates the phase at the selected points. The polynomial's constant term must be set to be 0 to guarantee that *I*_*k*_mr__(*x*_*c*_, 0) remains unchanged. For example, for the first direction, at selected values of *k*_D1_ the polynomial looks like
  (22) $$∠I_{k_{\text{mr}}}(x_{c},(k_{\text{D}1},0,0))\approx r_{\text{D}1}(k_{\text{D}1})≐a_{m}{\left(k_{\text{D}1}\right)}^{m}\;+a_{m\;-1}{\left(k_{\text{D}1}\right)}^{m-1}+\ldots +a_{1}k_{\text{D}1}$$
  as demonstrated in Figure 2.
5. Apply the same phase correction systematically to the entirety of the data along the *l*th direction at each of the other dimensions, at all of the pixel locations. For example in the first direction, *k*_D1_, update *I*_*k*_mr__ to be equal to 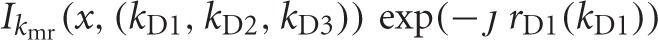 for all *k*_D2_, *k*_D3_ and *x*.
6. Repeat steps 2–5 for the remaining directions.

**Figure 3:**
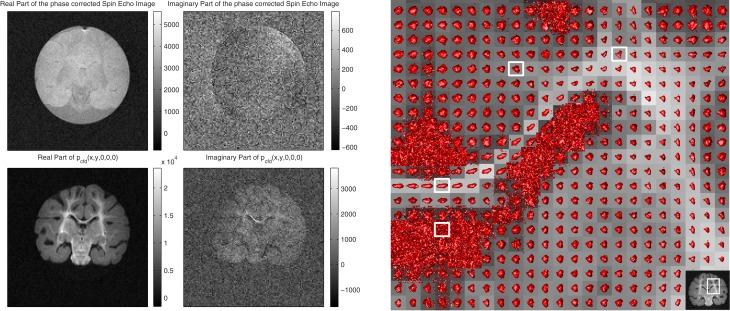
**On the left, the fixed baboon brain images acquired in the anatomical-coronal plane.** On the top row, the real and imaginary parts of *I*_*k*_mr__(*x*, 0) and on the bottom row, *P*_cfd_(*x*, 0) are displayed. The imaginary part is approximately 10% of the real part in both cases. **On the right**, full representation of *P*_cfd_ with isosurfaces (${\bar{P}}_{\text{cfd}}=0.17$, see section 3.1) around CC and EC junction. Starting from left bottom going clockwise, the sample pixels are from cerebro-spinal fluid (CSF), CC, white matter (WM) and CC, and EC junction, respectively (see also Figure 5).

The algorithm transfers the signal to the real channel by preserving its energy as the phase corrected spin-echo image without diffusion gradients, *I*_*k*_mr__(*x*, 0). The distribution of the magnetic moments with low mobility in all three directions [i.e., *P*_cfd_(*x*, 0)] shows the result of the transfer in Figure 3 for the sample described in section 2.4.

In *P*_cfd_(*x*, 0), areas with high level of organization inducing low mobility, such as the corpus callosum (CC), the external capsule (EC), the mid-brain and the pons, appear brighter. The image is not an anisotropy map, e.g., mineral oil would appear brighter than water due to a smaller diffusion coefficient despite both liquids being isotropic. Spin-echo image is more blurred because it is a low pass filtered version of *P*_cfd_:

(23) $$\begin{array}{l} I_{k_{\text{mr}}}^{\text{complex}}(x,\;k_{\text{D}})={ℱ}_{k_{\text{mr}}}^{-1}\{S_{\text{cfd}}(k_{\text{mr}},\;k_{\text{D}},\;k_{\text{rw}})\}\Rightarrow I_{k_{\text{mr}}}(x,\;0)\\ \;=\frac{2\pi }{\gamma }\int P_{\text{cfd}}^{\text{total}}(x,\;W)\;dW \end{array}$$

(see Appendix C).

CFD phase correction algorithm outperformed the fitting of the phase values up to the fourth degree multinomials in ${\mathbb{R}}^{3}$. The reasons behind this outcome, which will provide information about DW–MR signal artifacts, as well as inclusion of different functions for corrections will be investigated in the future.

### 2.3. CFD-MRI sampling and windowing

Whereas the standard MRI field of view (FOV) calculations (Haacke et al., 1999) are used for *k*_mr_-space, the infinite bandwidth in *k*_D_-space due to *P*_cfd_'s finite support in *W*^d^-space (originating from finite length displacements) falls beyond the reach of the gradient hardware's limits for small diffusion gradient duration and separation times (δ and Δ, respectively in Figure 1). Even with a powerful gradient system, a large magnitude of *k*_D_ causes substantial signal uncertainties due to an increasing performance deterioration as the power requirements push the hardware to its limitations.

**Figure 4:**
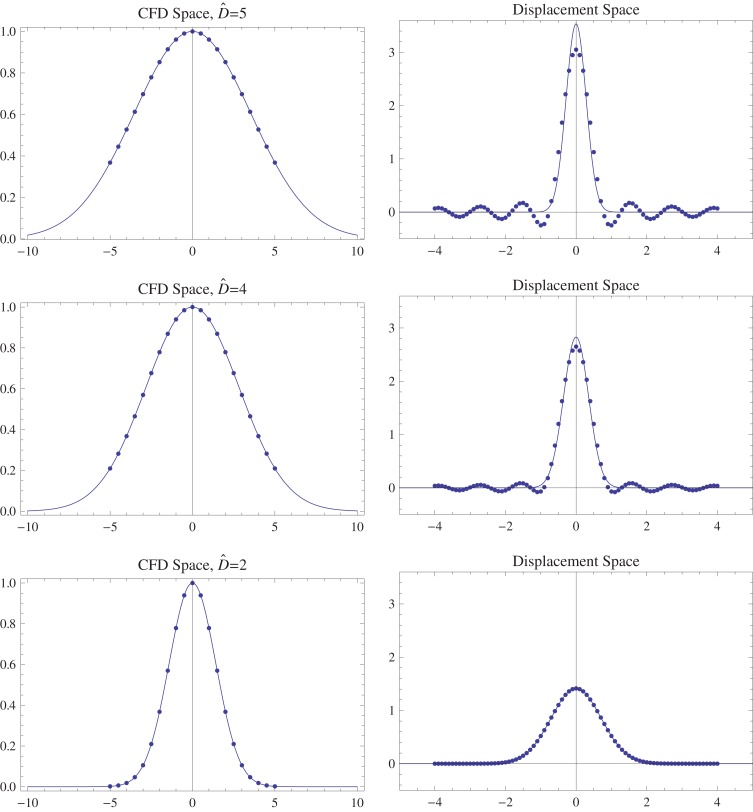
**Effects of fitting the bandwidth within the field of view (FOV) in the Fourier space on the **left**, with the covariance of the Gaussians given as $\hat{D}={\left(\delta^{2}(\Delta -\delta /3)D\right)}^{-1}$.** The sampling is done in the fixed interval, [‒5, 5], of the Fourier Space followed by reconstruction on the right using the discrete Fourier transform. Theoretically this is equivalent to convolution with the sinc function (see Brigham, 1988) in physical space. The ripple effects created by sinc lobes diminish on the left as the Gaussian falls into the FOV with increasing δ, Δ but constant *D*.

With such a hardware constraint, in order to reduce ripple effects caused by truncation, *P*_cfd_'s bandwidth (i.e., *S*_cfd_'s support) is shrunk by increasing δ and Δ causing the dispersion (covariance) of the displacement integral *W*^d^ (and therefore *P*_cfd_'s support) to increase. This is directly visible in the *special case* of Brownian Motion characterized by the diffusion tensor *D* in Figure 4. *P*_cfd_ and *S*_cfd_ are zero mean Gaussians with covariances, respectively equal to (see Appendix A)

(24) $$E[W^{\text{d}}{\left(W^{\text{d}}\right)}^{T}]=b_{t}\;D\text{ and }{\left(b_{t}\;D\right)}^{-1}\text{ where }b_{t}≐\delta^{2}(\Delta -\delta /3)$$

(see Özcan (2009, 2010a) because the Fourier transform of a Gaussian with a covariance matrix $\hat{D}$ is also a Gaussian with covariance ${\hat{D}}^{-1}$:

(25) $$ℱ\left\{\exp\left({\left(W^{\text{d}}\right)}^{T}{\hat{D}}^{-1}\;W^{\text{d}}\right)\right\}\sim \exp\left(k_{\text{D}}^{T}\;\hat{D}\;k_{\text{D}}\right).$$

The procedure is graphically displayed in Figure 4 also emphasizing the effect of ripples on the small values of *P*_cfd_ which are especially important in revealing microstructure as explained in section 3.1.

The second sampling criterion is an appropriate sampling rate i.e., sufficient number of points in *k*_D_-space to prevent aliasing artifacts on *P*_cfd_. This is constrained by the time available for acquisitions as each point in *k*_D_-space requires the scan time of the entire *k*_mr_-space.

### 2.4. Experimental setup and analysis methods

A fixed baboon brain immersed in 4% paraformaldehyde was used for the experiments. The primate was prematurely delivered on the 125th day and sacrificed on the 59th day after delivery. All animal husbandry, handling, and procedures were performed at the Southwest Foundation for Biomedical Research, San Antonio, Texas. Animal handling and ethics were approved to conform to American Association for Accreditation of Laboratory Animal Care (AAALAC) guidelines. Further details of the preparation are described in Kroenke et al. (2005).

The experiments were carried out on a 4.7 Tesla MR scanner (Varian NMR Systems, Palo Alto, CA, USA) with a 15 cm inner diameter gradient system, 45 Gauss/cm maximum gradient strength and 0.2 ms rise time using a cylindrical quadrature birdcage coil (Varian NMR Systems, Palo Alto, CA, USA) with 63 mm inner diameter. CFD–MRI data were obtained using the standard pulsed-gradient spin-echo multi-slice sequence. The *k*_mr_-space was sampled to result in images of 128 × 128 pixels with a FOV 64 × 64 mm^2^ and 0.5 mm slice thickness. The *k*_D_-space was sampled in a uniformly spaced Cartesian grid in a cube [−30, 30 Gauss/cm]^3^ with 6 Gauss/cm sampling intervals at each dimension resulting in 11× 11 × 11 voxels. The repetition time *T*_*R*_ = 1 s, echo time *T*_*E*_ = 56.5 ms, diffusion pulse separation time Δ = 30 ms and diffusion pulse duration δ = 15 ms were used.

The data were transferred to a two quad core 2.3 GHz Intel Xeon® cpu and 8 GB memory Dell Precision Workstation 490 running Windows XP® 64-bit operating system. The DWI data were placed in a 5-dimensional array in the computer memory and the discrete Fourier transform (DFT) was computed along with the phase corrections. In-house Matlab® (Mathworks, Natick, MA, USA) programs were used for all of the computations and to display the graphics and maps.

## 3. Results

### 3.1. Visualization of the CFD distribution

**Figure 5:**
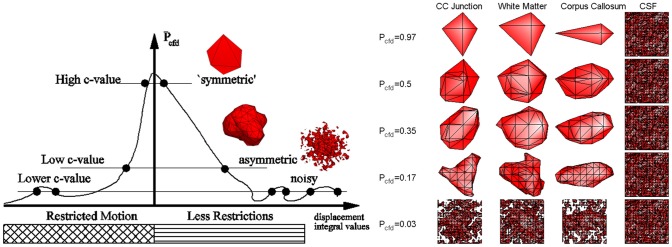
**On the left, one dimensional graphical representation of the choice of *c*-value.** The drawing below the horizontal axis displays the structure of the sample in the infinitesimal volume element [‒*dx*, *dx*] as seen by the magnetic moments from their initial position. The right side of the sample has less restrictive properties as on the boundary of tissue with a liquid, such as CC and cerebrospinal fluid (CSF). The noiseless isosurfaces consist of two points shown as dots on the graph. Low *c*-values correspond to the magnetic moments with longer travel paths providing more structural information than high *c*-values. However, too low values create noisy and disconnected isosurfaces represented with more than two points on the drawing. **On the right**, isosurfaces from different pixels in the baboon brain marked in Figure 3 demonstrate the effect of *c*-value on the information content from top = insufficient to bottom = noisy (see the text for the CSF column).

The joint distribution's high-dimensionality [e.g., two dimensions for position in regular MR images (*l*_mr_ = 2), plus three more for displacement integrals] creates a visualization challenge which is addressed herein by using *P*_cfd_(*x*, 0) as the background image. Furthermore, the isosurface^3^ of normalized *P*_cfd_,

$${\bar{P}}_{\text{cfd}}(x,\;W^{\text{d}})=\frac{P_{\text{cfd}}(x,\;W^{\text{d}})}{P_{\text{cfd}}(x,\;0)}$$

is overlayed on the pixel at location *x*, as in Figure 3. For the sake of an objective assessment, the isosurfaces are defined using a common level value *c* (0 < *c* ≤ 1),

$$\left\{W^{\text{d}}\in {\mathbb{R}}^{3}:{\bar{P}}_{\text{cfd}}\left(x,\;W^{\text{d}}\right)=c\right\},$$

over all locations. The key point is the choice of an appropriate *c*-value that will reveal the outskirts of *P*_cfd_ corresponding to the small number of “scout” magnetic moments that travel further away thereby portraying the microstructure of the environment. In summary,

1. Too high values do not provide enough structural information (see first rows in Figure 5).
2. The appropriately informative value depends on the properties of the motion (thus of the microstructure) at a given location (compare columns of Figure 5, right side).
3. Too low values force the isosurfaces to become extremely noisy (see last row of Figure 5).

As the motion in highly organized tissue is less dispersed (i.e., a smaller support for *P*_cfd_ which implies a larger support for *S*_cfd_ thereby causing bigger truncation effects), increasing the diffusion gradient times δ and Δ in section 2.3 will create almost “flat” displacement integral distributions at an isotropic medium like CSF. In this case, the small valued distribution [caused by constant integral value ∫ *P*_cfd_(*x*, *W*^d^) *dW*^d^ = number of particles] is susceptible to noise, creating noisy isosurfaces of Figure 5 for all level values. In contrast, for the experiments conducted with an isotropic (water) phantom at much lower δ and Δ values, the isosurfaces were spheres for a wide range of *c*-values (not shown).

**Figure 6:**
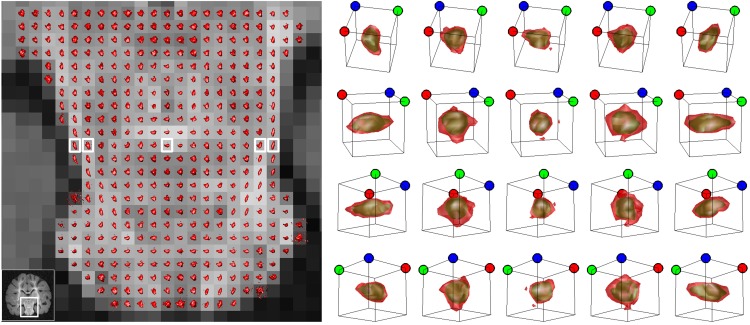
**The isosurfaces (${\bar{P}}_{cfd}=0.14$) on the pons and the mid-brain.** Each of the boxes indicate an isosurface presented, respectively on the right. Each column presents the same isosurface from different view angles. The dots on top of the frames are placed for orientative purposes. The surfaces are not necessarily ellipsoids and they have mostly an asymmetric structure. The outer red surface is the set ${\bar{P}}_{\text{cfd}}=0.14$ and the green surface is ${\bar{P}}_{cfd}=0.21$. The red surface envelops the green one.

Figure 6 presents different isosurfaces that elucidate the tissue structure on the pons and the mid-brain of the fixed baboon brain sample described in section 2.4. The tracts on the left and right side of the mid-brain are visible with the ellipsoidal looking isosurfaces. Five isosurfaces from the same row of pixels marked on Figure 6 are displayed on Figure 6 corresponding to two different *c*-values. The green isosurfaces with larger level values are smoother and less informative than the red ones with smaller *c*-values. Different viewpoints at each row of Figure 6 emphasize that the isosurfaces are 3-D objects. The figure demonstrates one of the challenges of presentation: the displacement integral values must be considered in ${\mathbb{R}}^{3}$ to grasp the complete information offered by CFD–MRI.

Overall, the isosurfaces are not constrained to given forms like Gaussians, spherical harmonics or to any expansions. In fact, they are typically not even symmetric. They are structureless, general and direct.

**Figure 7:**
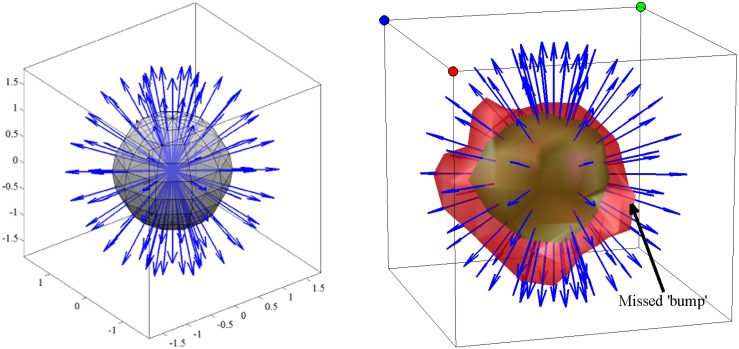
**An example of a set of rays from the origin along which the distribution function is integrated to obtain orientation distribution function.** As the rays disperse with increasing distance from the origin the points describing the displacement of a smaller number of particles contributes less and less to the numerical integration due to sparse sampling on both surfaces. The encapsulation of the isosurfaces on the right with larger level values by the smaller ones in Figure 6 shows the information that would be missed with numerical radial integration. The utilization of isosurfaces is more informative as discussed in Figure 5.

Isosurface visualizations constitute only one method to present the high dimensional information obtained from CFD–MRI. Another example is the dimension reduction by means of computing the so called orientation distribution function (ODF) (Wedeen et al., 2005) obtained from radial integrals. However, for CFD–MRI the ODF raises the concern of inadequately presenting the “outskirts” of the *P*_cfd_ because the dispersion of the outgoing rays shown in Figure 7 jeopardizes the inclusion of the values further away from the origin (see also Figure 6).

New methods, additional elaborate schemes such as color coding for representation of three dimensional functions aimed at displaying relevant microstructural information of CFD are left for future studies.

## 4. Discussion

### 4.1. Comparison with existing methods

From a fundamental point of view, guided by the microstructure that surrounds them, the molecules are displaced due to thermal energy whether they are in the scanner or not. All existing DW–MR methods are designed with the same goal in mind: the reconstruction of the propagator^4^ that describes the displacement of the magnetic moment from the DW–MR signal.

**Figure 8:**
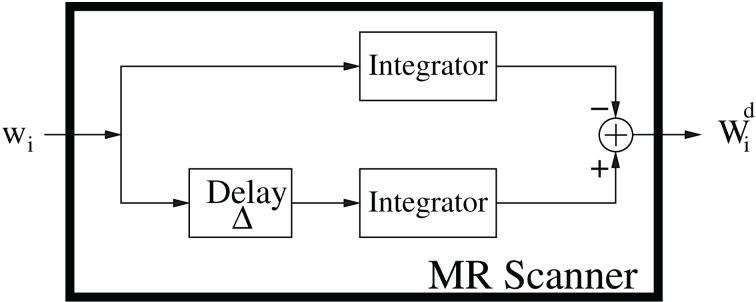
**The DW–MRI signal from a signals and systems perspective (Özcan, 2010b) describing the MRI observables of the diffusion phenomenon.** The input is the magnetic moment displacement *w*_*i*_ and the output is the displacement integral, *W*^*d*^_*i*_, defined in Equation (8).

However, as CFD–MRI demonstrates, from a systems science perspective the MRI scanner acts as a time-delay linear system with the input *w*_*i*_ [displacement in Equation (4)], and the output *W*_*i*_ [displacement integral in Equation (8)], in Figure 8. The parameters Δ and δ define the delay and filter parameters. Special attention is paid in CFD–MRI to isolate the Fourier variable, *k*_D_ = *G*_D_ from these parameters in contrast to the *q*-space variable (Callaghan, 1991): *q* = (2π)^−1^ γ δ *G*_D_ and the *b*-value of DTI: *b* = γ^2^ ‖ *G*_D_ ‖^2^_2_ δ^2^ (Δ − δ/3) (see Appendix A).

The inverse problem of obtaining the propagator from the distribution of the displacement integrals is singular because of the one-to-many relationship between the displacement and its integral:

(26) $$W_{i}^{\text{d}}=\int_{t_{d3}}^{t_{d4}}w_{i}(\tau )\;d\tau -\int_{t_{d1}}^{t_{d2}}w_{i}(\tau )\;d\tau \;\to \;w_{i}(t),$$

because all of the displacements with the same low frequency content in time are mapped to the same displacement integral value. This statistical accumulation prevents the determination of the propagator in a general environment from the distribution of displacement integrals^5^).

Existing methods' attempts to estimate the propagator relies on the narrow pulse approximation by assuming negligible pulse duration (δ = *t*_*d*2_ − *t*_*d*1_ = *t*_*d*4_ − *t*_*d*3_ in Figure 1) compared to pulse separation time, Δ, i.e., (δ << Δ) ⇒ (δ → 0) specifically in (Δ−δ/3) (Callaghan, 1991, p. 342). Under this short integration time assumption, in Wedeen et al. (2005) it is further argued that the approximation, *W*^d^_*i*_ ≈ (*x*_*i*_(*t*_*d*3_) − *x*_*i*_(*t*_*d*1_)) δ = (*w*_*i*_(*t*_*d*3_) − *w*_*i*_(*t*_*d*1_)) δ, is plausible. Although by the intermediate value theorem and the sample path continuity of the Brownian motion (Shiryaev, 1995), the values of each integral in *W*^d^_*i*_ are attained at a time point within the respective integration intervals, [*t*_*d*1_, *t*_*d*2_] and [*t*_*d*3_, *t*_*d*4_], the nowhere-differentiability of the sample paths (Shiryaev, 1995) implies that the intermediate time points satisfying the equality are not fixed as *t*_*d*1_ and *t*_*d*3_, but are themselves random variables. In consequence, without the inference of the displacements at fixed time points the propagator cannot be reconstructed.

Moreover, elaborate derivations carried in Wedeen et al. (2005) to model the propagator as a conditional probability, *p*(*x*(*t*_*d*3_)|*x*(*t*_*d*1_)), describing a Markovian process raise concerns specially in environments such as biological tissue since the particle's past collisions with microstructure guides its future displacements. In fact, while it violates the conditions of Wiener process (see Appendix A), this displacement memory provides the inference of the microstructure by way of affecting the displacements and consequently their displacement integral distributions. Accordingly, in CFD–MRI is indifferent to memory properties by modeling *P*_cfd_ as a joint distribution function of random variables instead of the conditional probability of a stochastic process.

A summary of CFD–MRI's detailed comparison with existing methods (Özcan, 2011; Özcan et al., 2012) is presented below for completeness of exposure. Namely, there exits two avenues for the path from DW–NMR to DW–MRI in the literature:

1. **Model matching methods** initiated by diffusion tensor imaging (DTI) (Basser et al., 1994; Mattiello et al., 1994) and further expanded with high angular resolution DW imaging (HARDI) (Frank, 2001), composite hindered and restricted model of diffusion (CHARMED) (Assaf and Basser, 2005), diffusion orientation transform (DOT) (Özarslan et al., 2006), two versions of the generalized DTI (GDTI) (Özarslan and Mareci, 2003; Liu et al., 2004), and diffusional kurtosis imaging (DKI) (Jensen et al., 2005).
2. **Spectral methods** originating from Callaghan's *q*-space (Callaghan et al., 1988) followed by the diffusion spectrum imaging (DSI) (Wedeen et al., 2005) and Q-ball imaging (Tuch, 2004).

With the exception of the GDTI presented in Liu et al. (2004) [see also the discussion in Özarslan et al., (2009)], all of the DW–MRI methods estimate symmetric quantities. The model matching methods project the data onto symmetric structures, such as ellipsoids in DTI or spherical harmonics in HARDI. The spectral methods use the magnitude of the signal in the Fourier transform resulting in symmetric functions (see section 2.2). It is difficult to imagine that molecular motion in a biological environment populated with different types of fluids, barriers and tissue would be symmetric at any given location, e.g., at the fiber junctions. Symmetry or lack of it ought to be determined by the data free of any constraints imposed by the model as in the implementation of CFD–MRI's unconstrained structure.

The Fourier relationship between signal and joint distribution function provides a complete understanding of model matching methods. The methods start by applying DFT to the data in the first *l*_mr_ (imaging) dimensions. Thus, in the analysis of model matching methods the first *l*_mr_ independent variables are *the physical location*. The three remaining untransformed variables are the independent variables of the Fourier reciprocal of displacement integral space, i.e., they are *in the Fourier domain*. The goal of model matching methods is to fit the preferred model to the displacement distribution function's Fourier transform, sampled at the (vector) values defined by the diffusion sensitizing gradients. In the case of Brownian motion this mixed variable (physical and frequency variables) approach is applicable because the diffusion coefficient *D* can be directly estimated from the Fourier domain by Equations (24) and (25). The mixed space, which works well for DW–NMR signal peak attenuation, is translated to DW–MRI at each position *x* by

(27) $$\left|I_{k_{\text{mr}}}^{\text{complex}}(x,\;k_{\text{D}})\right|=\left|I_{k_{\text{mr}}}^{\text{complex}}(x,\;0)\right|\exp(-H(k_{\text{D}}))$$

where *I*_*k*^complex^_mr__ is given in Equation (18) and the function *H* defines the model, e.g., the quadratic form of DTI, spherical harmonics of HARDI or higher tensor expansions of GDTI [see Özcan et al. (2012) for a detailed exposition]. In a sense, these methods' aim could be summarized as expanding the Fourier portion of the mixed signal space. The basic example with a single term in the expansion is DTI for which:

(28) $$H(k_{\text{D}})=\gamma^{2}\;b_{t}\;k_{\text{D}}^{T}\;D\;k_{\text{D}}$$

where the calculation of *b*_*t*_ from the PDE approach in Özcan (2010a) and covariance of displacement integrals in Appendix A resulted in the same value: δ^2^ (Δ − δ/3). In CFD parlance, by the Fourier relationship between Gaussians in Equation (25), the diffusion quadratic form, *D*, is estimated in *k*_D_-space, without recourse to a Fourier transform because of its direct appearance in Equation (28) rather than its inverse, *D*^−1^, in the Gaussian of motion space. The coefficient matrix defined by carefully selected vectors in *k*_D_-space that satisfy the invertibility conditions (Özcan, 2005) is used for the estimation of *D* in the linear algebraic framework of symmetric matrices (Papadakis et al., 1999; Özcan 2010a) [also refer to Özcan (2010a) and Özcan (2012) for the correspondence with the *B*-matrix formulation of Basser et al., (1994)].

The magnitude-based Fourier relationship presented in *q*-space methodology (Callaghan et al., 1988) is the origin of spectral methods. In Callaghan's book (Callaghan, 1991), parallel to the historical development of DW models, the theory is first developed for NMR experiments [see Callaghan, (1991, Chap. 6)] using polarized neutron scattering analogy. However, the translation from NMR to MRI is presented [see asserting without proof that the imaging and displacement portions of the signal are separable [see Callaghan, (1991, Chap. 8, pp. 440)]. The derivations of section 2.1 demonstrate that this is not the case.

**Figure 9:**
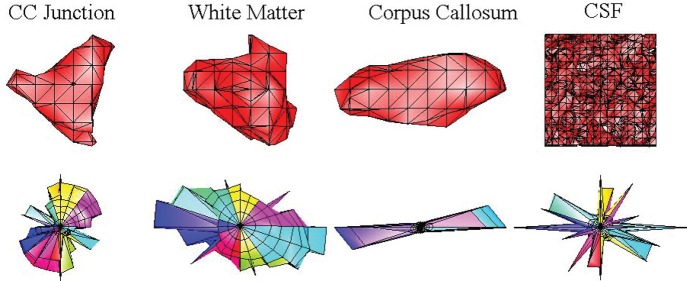
**The comparison of *P*_cfd_ (Özcan, 2011) (first row) with the diffusion spectrum imaging orientation distribution function (DSI–ODF) (second row).** Both functions are calculated from the same data on the right junction of the corpus callosum (CC) and the external capsule (EC), specifically from the pixels marked on the Figure 3. The isosurface (${\bar{P}}_{\text{cfd}}=0.17$) captures the asymmetric structure of the fiber crossings while the ODF is constrained to be symmetric for all of the pixels. Note that in CSF, ODF detects structure which is not present in reality as indicated by CFD.

In addition, by the affine dependence of *k*_rw_ on *k*_mr_ CFD derivations show that the inseparability partially accounts for the non-Hermitian nature of the *S*_cfd_. Taking the magnitude of the DW–MRI signal, as in the case of DSI (Wedeen et al., 2005), does not count as a phase correction. Figure 9 demonstrates that by preserving Hermitian property, CFD–MRI captures correctly the crossing fibers at the junction of the CC and EC.

### 4.2. Conclusion and future studies

In the biomedical imaging modalities' grand aim of biomarker capability establishment, the discovery path for CFD–MRI passes through the distribution function:

$$S_{\text{cfd}}\to P_{\text{cfd}}\to \text{Biologicalproperties}.$$

With *P*_cfd_ in the middle, both sides of the path present themselves with important challenges.

First and foremost, in DW–MRI, the displacements without reference to initial positions [see Equation (6)] prevent the inference of microstructure position. For example, the distribution function of the biological phantom (Özcan et al., 2011) constructed with two crossing rat trigeminal nerve fibers is always in the form of two crossing bars across the origin regardless of the nerves' position as long as their relative angle is kept the same. Also in the same phantom, the agar gel (isotropic component) appears as a sphere around the origin of *P*_cfd_ domain without the possibility of identifying its location. As the distributions from various types of microstructural components accumulate around the origin, the discrimination level of overlaps, more prominent with increasing biological tissue complexity, directly defines the sensitivity and specificity for microstructure changing pathologies. The important goal is the assessment of the theoretical aspects of the distribution function in order to understand whether it can detect in a timely manner, e.g., before significant disease progression, those changes. The determination of biophysical conditions behind the asymmetry (see also Özarslan et al., 2008; Özarslan, 2009) in the distribution functions is also part of the same goal.

However, the absence of analytical descriptions for *P*_cfd_ even in simple environments requires the investigations to be conducted with numerical simulations (Özcan et al., 2011) of particle motion within carefully designed geometries (Landman et al., 2010) and locally variable diffusivities. Along with numerical phantoms mimicking biological ones (Özcan et al., 2011), histopathological information is also being used for interpretation and validation (Budde and Frank, 2012; Budde and Annese, 2013). Additionally varying the time parameters δ and Δ will exploit the filtering effects caused by the displacement integral that will determine whether further information extraction is possible by expanding data acquisition with an appropriate set of parameter values.

On the other hand, on the discovery path's initiation by CFD–MRI signal formation, the re-establishment of Hermitian symmetry requires, in addition to the theoretical reasons presented herein, the analysis and quantification of Hermitian disruptive artifacts and systematic conditions in real data. Constructed by initially experimenting with elementary phantoms (e.g., water and mineral oil), this signal model expansion is necessary for the development of more elaborate systematic phase corrections, possibly by utilizing complex analysis theory. Specifically, accurate estimation of the pertinent Fourier transform of *P*_cfd_ from real data points in a clinical setup is targeted by the adaptation of CFD–MRI to fast sequences^6^, such as echo planar imaging (EPI) prone to Eddy current artifacts. The model will be expanded up to the point of reaching only minimal incremental improvements with new phase correction algorithms. Thereafter, relying on the residuals' content, which is free from displacement effects consequent to the application of system-wide uniform phase corrections^7^, more effective biomarker construction would be possible by the inclusion of extra information such as tissue susceptibility (Liu et al., 2013). Likewise, on a larger scale CFD–MRI's general aim is to improve outcomes of multimodal imaging, e.g., in prostate cancer strategies (Turkbey and Choyke, 2012).

### Conflict of interest statement

The author declares that the research was conducted in the absence of any commercial or financial relationships that could be construed as a potential conflict of interest.

## Footnotes

^1^The refocusing lobe of the slice select gradient after the RF pulse denoted by SS in Figure 1 adjusts ϕ_slice_ to be constant throughout the slice in the slice select direction.

^2^The unit for *k*_mr_ is $\frac{\text{magnetic field}\times \text{time}}{\text{distance}}$ but for *k*_D_ and *k*_rw_ it is $\frac{\text{magnetic field}}{\text{distance}}$. They are both in accordance with the units of the position and the displacement integrals in Equation (13). After *k*_cfd_ is multiplied by the gyromagnetic ratio γ, with unit (magnetic field × time)^−1^, the product becomes unitless in Equation (14).

^3^Another approach in the literature is to present the value of the function over a sphere (Tuch, 2004; Wedeen et al., 2005). However, uniqueness is lost in this representation as demonstrated by these functions: *f*(*x*, *y*, *z*) = *x*^2^ + *y*^2^ + *z*^2^, *h*(*x*, *y*, *z*) = (*f*(*x*, *y*, *z*))^2^, which have the same value on the unit sphere but different isosurfaces.

^4^Also named nuclear spin self-correlation function (Callaghan et al., 1988) and translation probability (Cory and Garroway, 1990).

^5^One exception is the basic case of isotropic diffusion analyzed in Appendix A where the diffusion coefficient that describes the Gaussian propagator can be recovered using the adjustment factor for the displacement integral covariance namely *b*_*t*_ in Equation (24) and the *b*-value in DTI formulation.

^6^Acquisition time is shortened with reduced sampling schemes aimed at specific goals, e.g., compressed sensing for tractography (Landman et al., 2012) at the expense of some information loss.

^7^Instead of pixel by pixel corrections that would completely eliminate the imaginary part in Figure 3.

## Glossary

*w*_*i*_: *i*th magnetic moment's displacement from its initial position *x*_*i*_(*t*_0_).
*W*^d^: The difference of magnetic moment displacement integrals during the periods diffusion (d) gradients are turned on. Its distribution is the main quantity of interest providing information about microstructure. Joined with the other displacement integrals during acquisition and rewind times, *W*^acq^, *W*^rw^, respectively, it forms the total displacement integral: $W=(W^{\text{d}},W^{\text{rw}},W_{1}^{\text{acq}})\in {\mathbb{R}}^{7}$.
*P*_cfd_(*x*, *W*^d^): CFD joint distribution function of the number of magnetic moments with initial position *x* possessing displacement integral values of *W*^d^. It is obtained by marginalizing *P*^total^_cfd_(*x*, *W*).
*S*_cfd_(*k*_mr_, *k*_D_, *k*_rw_): DW–MRI signal that comes out of the scanner which is equal to the Fourier transform of *P*^total^_cfd_. Theoretically it must be Hermitian symmetric since *P*^total^_cfd_ is real valued.
(*k*_mr_, *k*_D_): *k*_cfd_-space variables corresponding to imaging gradients (mr) and diffusion (D) gradients, respectively.
*k*_rw_: Rewinding (rw) frequency variable affine dependent on *k*_mr_. It cannot be sampled at 0 because of its dependence. For *P*^total^_cfd_ marginalization, phase corrections are applied to estimate *S*_cfd_ at *k*_rw_ = 0.
*I*_*k*^complex^_mr__(*x*, *k*_D_): Complex valued images obtained by taking the Fourier transform of *S*_cfd_ with respect to imaging frequency *k*_mr_.
*I*_*k*_mr__(*x*, *k*_D_): Image domain phase corrected *I*_*k*^complex^_mr__(*x*, *k*_D_).

## Appendices

### A. Second order statistics for displacement integrals of self diffusion

In this section, the covariance of displacement integrals is derived for the special case of particles executing Brownian motion. The following preliminaries are necessary to calculate the second order statistics for the displacement integrals of Equation (9). It is assumed for ease of notation below that *t*_*m*_ ≤ *t*_*m* + 1_.

The Wiener process (Shiryaev, 1995), *w*, which describes the diffusion in an isotropic homogenous medium, is sample path continuous, has *w*(0) = 0 and independent increments (i.e., Markovian property) with a normal distribution, meaning w(t)−w(s)~ℕ(0,(t−s)D) where *t* > *s* ≥ 0 and *D* > 0. Using these properties, the covariance of the displacement is:

(29) $$\begin{array}{l} E\left[w(t)\;w^{T}(s)\right]=E\left[\left(w(t)-w(s)+w(s))\;w^{T}(s\right)\right]\\ \;=E\left[w(s)w^{T}(s)\right]=\min(t,\;s)\;D. \end{array}$$

It is straightforward to show that the mean for the displacement integral processes is 0. The covariance in the case of a single interval is calculated as

(30) $$\begin{array}{c} E\left[\left(\int_{t_{1}}^{t_{2}}w_{i}(\tau )d\tau \right)\left(\int_{t_{1}}^{t_{2}}w_{i}^{T}(s)ds\right)\right]=\int_{t_{1}}^{t_{2}}\int_{t_{1}}^{t_{2}}E\left[w_{i}(\tau )w_{i}^{T}(s)\right]d\tau ds\\ =\int_{t_{1}}^{t_{2}}\int_{t_{1}}^{t_{2}}\min(\tau ,s)Dd\tau ds=\frac{1}{3}{\left(t_{2}-t_{1}\right)}^{2}(2t_{1}+t_{2})D, \end{array}$$

and for a non-overlapping interval:

(31) $$\begin{array}{l} \int_{t_{3}}^{t_{4}}\int_{t_{1}}^{t_{2}}E\left[w_{i}(\tau )\;w_{i}^{T}(s)\right]\;d\tau \;ds=\int_{t_{3}}^{t_{4}}\int_{t_{1}}^{t_{2}}\min(\tau ,\;s)D\;d\tau \;ds\\ =\frac{1}{2}(t_{4}-t_{3})(t_{2}^{2}-t_{1}^{2})D. \end{array}$$

Finally, using the formulas above, the time points of Figure 1, [*t*_1_ *t*_2_ *t*_3_ *t*_4_] = [*t*_*d*1_ *t*_*d*2_ *t*_*d*3_ *t*_*d*4_], and these substitutions,

$$\begin{array}{l} \delta =t_{2}-t_{1}=t_{d2}-t_{d1}=t_{4}-t_{3}=t_{d4}-t_{d3},\\ \Delta =t_{3}-t_{1}=t_{d4}-t_{d2}=t_{4}-t_{2}=t_{d3}-t_{d1}, \end{array}$$

the following is obtained for *W*^*d*^_*i*_:

(32) $$\begin{array}{l} E\left[W_{i}^{\text{d}}{\left(W_{i}^{\text{d}}\right)}^{T}\right]=[({\left(t_{2}-t_{1}\right)}^{2}(2t_{1}+t_{2})+{\left(t_{4}-t_{3}\right)}^{2}\left(2t_{3}+t_{4}\right))/3-(t_{4}-t_{3})(t_{2}^{2}-t_{1}^{2})]D\\ \;=\delta^{2}(\Delta -\delta /3)D. \end{array}$$

The scalar factor, δ^2^ (Δ − δ/3), defined as *b*_*t*_-value in Özcan (2009, 2010a), is a function of time directly related to *the measured quantity*, the displacement integrals. It is completely detached from the measurement parameters such as diffusion gradient strength, in contrast to the *b*-value used in the literature, *b* = γ^2^ ‖*G*_D_‖^2^_2_ *b*_*t*_. The dependence of the derivations on Markovian property restricts the validity of the calculations to isotropic samples where the diffusion is characterized by the diffusion coefficient, *D*, in the distribution of magnetic moment displacement *w*. For an isotropic homogenous medium, *D* is estimated from the displacement integral's (*W*^d^) covariance *b*_*t*_ *D* = δ^2^ (Δ − δ/3)*D* using DW–MRI acquisitions.

The rigorous treatment of the theory of the stochastic processes in Doob, (1990, p. 62) demonstrates that the sample paths of stochastic processes are Lebesgue integrable under certain conditions and these integrals are well defined random variables. Although a rigorous mathematical analysis, which proves that these conditions are met, is out of the scope of this manuscript, it is important to note that Equation (23) does not prove that the displacement integrals are Gaussian random variables. In this case, the central limit theorem does not apply because the displacement integrals of Equations (8, 9), are not the sums of independent variables since in the integral approximating sum

(33) $$\begin{array}{l} \int_{t_{0}}^{t_{1}}w_{i}(\tau )\;d\tau \approx \underset{N\to \infty }{\lim}\sum_{k=1}^{N}w_{i}(t_{0}+k\;d\tau )\;d\tau \\ \;(N\to \infty ,\;d\tau \to 0,\;N\;d\tau =t_{1}-t_{0}), \end{array}$$

*w*_*i*_(τ), rather than independent increments, *w*_*i*_(*t*) − *w*_*i*_(*s*), is present. A variant of central limit theorem for sums of dependent variables satisfying certain conditions (Shiryaev, 1995, p. 541) might provide the theoretical validation of this highly theoretical open problem.

### B. Derivation of the phase equation

#### B.1. Bloch equations and their solutions

The time evolution of the magnetization, ${\bar{m}}_{i}(t)\in {\mathbb{R}}^{3}$, that generates the MR signal is modeled by the Bloch equations:

(34) $$\frac{d{\bar{m}}_{i}(t)}{dt}=\gamma \;{\bar{m}}_{i}(t)\times B(x_{i},\;t)-\left[\begin{array}{ccc} \frac{1}{T_{2i}} & 0 & 0\\ 0 & \frac{1}{T_{2i}} & 0\\ 0 & 0 & \frac{1}{T_{1i}} \end{array}\right]{\bar{m}}_{i}(t)+\frac{1}{T_{1i}}\left[\begin{array}{c} 0\\ 0\\ m_{3i} \end{array}\right]$$

with *T*_1*i*_ and *T*_2*i*_ denoting spin–lattice and spin–spin relaxation constants, respectively. In the MR scanner the magnetic field as a function of time and position is given by

(35) $$B(x,\;t)=\left[\begin{array}{c} 0\\ 0\\ B_{0} \end{array}\right]+B_{1}^{m}(t)+\left[\begin{array}{c} 0\\ 0\\ G(x,\;t)\;\cdot \;x(t) \end{array}\right]$$

where *B*_0_ is the static (strong) magnetic field, *B*^*m*^_1_(*t*) is the radio frequency (RF) pulse modulated at the precession frequency of *B*_0_ and $G(x,t)\in {\mathbb{R}}^{3}$ describes the magnetic field gradients. The expression of the magnetic field simplifies further in the rotating frame:

(36) $$B(x,\;t)=B_{1}(t)+\left[\begin{array}{c} 0\\ 0\\ G\left(x,\text{ t}\right)\;\cdot \;x\left(t\right) \end{array}\right].$$

When the magnetic field gradients are turned on without the RF pulse the system matrix becomes

(37) $${\bar{m}}_{i}\times \left[\begin{array}{c} 0\\ 0\\ \omega (x_{i}(t)) \end{array}\right]=\left[\begin{array}{ccc} 0 & \omega (x_{i}(t)) & 0\\ -\omega (x_{i}(t)) & 0 & 0\\ 0 & 0 & 0 \end{array}\right]{\bar{m}}_{i}=A(t)\;{\bar{m}}_{i}.$$

with ω(*x*_*i*_(*t*)) = *G*(*x*_*i*_, *t*) · *x*_*i*_(*t*). As the time-dependent system matrix *A*(*t*) commutes with its integral ∫ *A*(τ)*d*τ, when the relaxation terms are negligible the solution of Equation (34) is obtained [see Rugh, (1995, pg. 59)] as:

(38) $${\bar{m}}_{i}(t)=\exp\left(\gamma \int_{t_{0}}^{t}A(\tau )d\tau \right)\;{\bar{m}}_{i}(t_{0})$$

by calculating the matrix exponential using Ω(*x*_*i*_(*t*), *t*_0_) = ∫^*t*^_*t*_0__ ω(*x*_*i*_(τ)) *d*τ = ∫^*t*^_*t*_0__ *G*(*x*_*i*_,τ)· *x*_*i*_(τ) *dτ*:

$$\exp\left(\text{​}\gamma \int_{t_{0}}^{t}A(\tau )d\tau \text{​}\right)=\left[\begin{array}{ccc} \cos(\gamma \Omega (x_{i}(t),\;t_{0})) & \sin(\gamma \Omega (x_{i}(t),\;t_{0})) & 0\\ -\sin(\gamma \Omega (x_{i}(t),\;t_{0})) & \cos(\gamma \Omega (x_{i}(t),\;t_{0})) & 0\\ 0 & 0 & 1 \end{array}\right].$$

Equation (38) defines a system with a rotating transverse magnetization component that can be written in a complex form . The corresponding first order differential equation is  with the solution

For the portion of the pulse sequence involving the RF pulses, in general (Bernstein et al., 2004) the magnetic moments are assumed to be immobile [i.e., *w*_*i*_(*t*) ≡ 0 in Equation (4)] thereby defining a constant system matrix that has a standard matrix exponential that solves the differential equation. Herein, however, the effect of the displacements specifically during the π-pulse results in a time varying system matrix. Without loss of generality, the π-pulse is applied in the direction^8^. [1, 0, 0]^*T*^ as in Hinshaw and Lent (1983), along with the slice select gradient, *G*_π_ = [0, 0, *g*_π3_], active only during the RF pulse. The RF pulse modulated at the same frequency corresponding to the center of the slice, *B*_0_ + *g*_π3_ *x*_*c*3_, becomes a constant vector, *B*_1_(*t*) = [*B*_1*a*_, 0, 0]^*T*^, in Equation (36). The system of equations in this rotating frame is

(41) $$m_{i}\times \left[\begin{array}{c} B_{1a}\\ 0\\ \omega_{\pi }(x_{i}(t)) \end{array}\right]=\left[\begin{array}{ccc} 0 & \omega_{\pi }(x_{i}(t)) & 0\\ -\omega_{\pi }(x_{i}(t)) & 0 & B_{1a}\\ 0 & -B_{1a} & 0 \end{array}\right]m_{i}=A(t)\;m_{i}$$

with^9^ ω_π_(*x*_*i*_(*t*)) = *G*_π_ · (*x*_*i*_(*t*_0_) + *w*_*i*_(*t*) − *x*_*c*_) = *g*_π3_ (*x*_3*i*_(*t*_0_) + *w*_3*i*_(*t*) − *x*_*c*3_). The procedure would be to solve Equation (41) and then express the solution in the initial rotating frame to obtain a phase component, Φ_π*i*_, that would be a function of the displacement integral and the initial position from the slice center.

However, the new system matrix, *A*(*t*), does not commute with its integral preventing the calculation of a matrix exponential formulating a convenient analytical solution as in Equation (40). With the rigorous analysis describing the exact manifestation of the RF pulse left to future studies, herein due to the large magnitude of the RF field in comparison to the magnetic field gradient, its effect will be approximated as a sign reversal of the phase. In this work, the phase change induced by the magnetic field gradient is considered as a factor that is automatically corrected by the phase correction algorithm of section 2.2.

On the other hand, the effect of the displacement induced by the RF slice select gradient (SS–EX in Figure 1) during the excitation period is systematic. As both the RF pulse and the magnetic field gradient remain fixed, statistically speaking, the phase of the total transverse magnetization is affected by the displacements in the same amount at each acquisition prior to the tipping. The position and displacement encoding occurs thereafter during the remainder of the pulse sequence (see also footnote 1 for the phase correction along the slice select direction within the slab) leaving the π/2-pulse out of the derivation of the phase equations below.

#### B2. Phase equation

For clarity of exposition, the calculations are carried out with the assumption of ideal gradient amplifiers creating rectangular shaped gradient pulses in the time domain and perfect linearity in physical space. In short, during the time interval when the amplifiers are powered on $G(x,t)=G\in {\mathbb{R}}^{3}$.

The derivation of Equations (5– 7) provided in Özcan (2012) is briefly presented in this section for reference purposes. Using the definitions of the variables in Figure 1, the evolution of the phase is described as:

1. First, imaging gradients for read out rewinding, $G_{\text{ro}}\in {\mathbb{R}}^{3}$, phase encoding, $G_{\text{pe}}\in {\mathbb{R}}^{3}$ and slice select $G_{\text{ss}}\in {\mathbb{R}}^{3}$ on the time interval [*t*_0_, *t*_rw_] are turned on. After these gradients are applied the phase and the magnetization become
  (42) $$\begin{array}{l} \Omega_{\text{rw}}=\int_{t_{0}}^{t_{\text{rw}}}(G_{\text{ro}}(\tau )+G_{\text{pe}}(\tau )+G_{\text{ss}}(\tau ))\;\cdot \;x_{i}(\tau )\;d\tau \\ \;=(G_{\text{ro}}+G_{\text{pe}}+G_{\text{ss}})\;\cdot \;\left(\Delta t_{\text{rw}}x_{i}(t_{0})+\int_{t_{0}}^{t_{\text{rw}}}w_{i}(\tau )\;d\tau \right)\\ \;=\Delta t_{\text{rw}}(G_{\text{ro}}+G_{\text{pe}}+G_{\text{ss}})\;\cdot \;x_{i}(t_{0})\\ \;+(G_{\text{ro}}+G_{\text{pe}}+G_{\text{ss}})\;\cdot \;\int_{t_{0}}^{t_{\text{rw}}}w_{i}(\tau )\;d\tau . \end{array}$$
2. The RF π-pulse between the diffusion gradient pulses, $G_{\text{D}}\in {\mathbb{R}}^{3}$, and the accompanying slice select gradient provide theoretical sign reversal of the phase. The slice select gradient's encoding of magnetic moment motion into the signal is expressed by Φ_π*i*_ (see Appendix B1). Since *x*_*i*_(*t*_*dk*_) = *x*_*i*_(*t*_0_) + *w*_*i*_(*t*_*dk*_), *k* = 1, …, 4; at *t* = *t*_*d*4_
  (44) $$\Omega_{\text{D}}=\Phi_{\pi i}+\int_{t_{d3}}^{t_{d4}}G_{\text{D}}\;\cdot \;x_{i}(\tau )\;d\tau -\int_{t_{d1}}^{t_{d2}}G_{\text{D}}\;\cdot \;x_{i}(\tau )\;d\tau$$
  (45) $$\begin{array}{l} =\Phi_{\pi i}+G_{\text{D}}\;\cdot \;\left((t_{d4}-t_{d3})x_{i}(t_{0})+\int_{t_{d3}}^{t_{d4}}w_{i}(\tau )\;d\tau \right)-G_{\text{D}}\;\cdot \;\left((t_{d2}-t_{d1})x_{i}(t_{0})+\int_{t_{d1}}^{t_{d2}}w_{i}(\tau )\;d\tau \right)\\ =\Phi_{\pi i}+G_{\text{D}}\;\cdot \;\left(\int_{t_{d3}}^{t_{d4}}w_{i}(\tau )\;d\tau -\int_{t_{d1}}^{t_{d2}}w_{i}(\tau )\;d\tau \right)+((t_{d4}-t_{d3})-(t_{d2}-t_{d1}))G_{\text{D}}\;\cdot \;x_{i}(t_{0}). \end{array}$$
  If the diffusion gradient times are equal, i.e., *t*_*d*4_ − *t*_*d*3_ = *t*_*d*2_ − *t*_*d*1_ = δ, then the last term in Equation (6) is equal to zero, erasing the influence of initial position from motion encoding part of the signal. This is the insight gained by using the formulation with displacement integrals, ∫ *w*_*i*_(τ) *d*τ, rather than the center of mass (COM) of random walk, ∫ *x*(τ) *d*τ, introduced in Mitra and Halperin (1995).
  The sign reversal affects also previously accumulated phase:
3. The last part of the sequence is where the data are collected:
  (47) $$\Omega_{\text{ro}}(t)=\int_{t_{\text{acq}}}^{t}G_{\text{ro}}\;\cdot \;(x_{i}(t_{0})+w_{i}(\tau ))\;d\tau =(t-t_{\text{acq}})\;G_{\text{ro}}\;\cdot \;x_{i}(t_{0})+\int_{t_{\text{acq}}}^{t}G_{\text{ro}}\;\cdot \;w_{i}(\tau )\;d\tau ,$$
  leading to  and Equations (5– 7).

### C. Total number of particles from CFD-MRI

The fundamental Fourier relationship of Equation (16), *S*_cfd_(*k*_mr_, *k*_D_, *k*_rw_) = ℱ{*P*^total^_cfd_}(*k*_mr_, *k*_D_, *k*_rw_) establishes the relationship between the standard MR image space and the higher dimensional CFD space. By Equation (16)  and by Equation (18), *I*_*k*^complex^_mr__(*x*, *k*_D_, *k*_rw_) = ℱ^−1^_*k*_mr__{*S*_cfd_(*k*_mr_, *k*_D_,*k*_rw_)}, the image obtained without the diffusion encoding magnetic field gradients is

(52) $$=\int^{\text{​}}P_{c\text{fd}}^{\text{total}}(x^{\prime },\;W)\text{δ}\left(\frac{\text{γ}}{2\pi }(x^{\prime }-x)\right)\;dx^{\prime }\;dW$$

(53) $$=\frac{2\pi }{\gamma }\int P_{\text{cfd}}^{\text{total}}(x,\;W)\;dW$$

(54) ~total number of particles at x.
